# Accumulation and Effect of Silver Nanoparticles Functionalized with *Spirulina platensis* on Rats

**DOI:** 10.3390/nano11112992

**Published:** 2021-11-07

**Authors:** Ludmila Rudi, Inga Zinicovscaia, Liliana Cepoi, Tatiana Chiriac, Alexandra Peshkova, Anastasia Cepoi, Dmitrii Grozdov

**Affiliations:** 1 Institute of Microbiology and Biotechnology, 1 Academiei Str., 2028 Chisinau, Moldova; rudiludmila@gmail.com (L.R.); lilianacepoi@yahoo.com (L.C.); chiriac.tv@gmail.com (T.C.); anastasiacepoi@gmail.com (A.C.); 2 Joint Institute for Nuclear Research, 6 Joliot-Curie Str., 141980 Dubna, Russia; peshkova.alexandra92@gmail.com (A.P.); dsgrozdov@rambler.ru (D.G.); 3 Horia Hulubei National Institute for R&D in Physics and Nuclear Engineering, 30 Reactorului Str., MG-6 Bucharest Magurele, Romania; 4 Institute of Chemistry, 3 Academiei Str., 2028 Chisinau, Moldova

**Keywords:** *Spirulina platensis*, silver nanoparticles, hematological indices, biochemical parameters, neutron activation analysis

## Abstract

The effect of unmodified and functionalized *Spirulina platensis* biomass silver nanoparticles on rats during prolonged oral administration was assessed. Silver nanoparticles were characterized by using transmission electron microscopy, while their uptake by the biomass was confirmed using scanning electron microscopy and energy dispersive analysis. The content of silver in the different organs of rats after a period of administration (28 days) or after an additional clearance period (28 days) was ascertained by using neutron activation analysis. In animals administrated with the unmodified nanoparticles, the highest content of silver was determined in the brain and kidneys, while in animals administrated with AgNP-Spirulina, silver was mainly accumulated in the brain and testicles. After the clearance period, silver was excreted rapidly from the spleen and kidneys; however, the excretion from the brain was very low, regardless of the type of nanoparticles. Hematological and biochemical tests were performed in order to reveal the effect of nanoparticles on rats. The difference in the content of eosinophils in the experimental and control groups was statistically significant. The hematological indices of the rats did not change significantly under the action of the silver nanoparticles except for the content of reticulocytes and eosinophils, which increased significantly. Changes in the biochemical parameters did not exceed the limits of normal values. Silver nanoparticles with the sizes of 8–20 nm can penetrate the blood–brain barrier, and their persistence after a period of clearance indicated the irreversibility of this process.

## 1. Introduction

Nanotechnology has been proven as one of the most dynamic fields of modern science. Nanomaterials are increasingly common in human life, and nanoparticles with unique properties have perfected many sectors of production and consumption, including medicine, where they are used for imaging and drug administration [1]. Silver nanoparticles (AgNPs) are among the most available nanomaterials produced on an industrial scale for various fields of application. AgNPs are used in the textile industry in the production of dyes, detergents, cosmetics, food packaging, etc. [2,3]. These nanoparticles have also been widely applied in medicine as antibacterial, antifungal, antiviral, anti-inflammatory, anti-angiogenic, and anticancer agents [4]. Their antimicrobial properties are considered to be a solution to the problem of multidrug-resistant bacteria [5,6].

The presence of nanoparticles in consumer products and their use in daily life can be associated with the risk of developing toxic effects on living organisms, including humans.

Nowadays, some mechanisms defining the toxicity of AgNPs are already known. In the presence of nanoparticles, the production of reactive oxygen species (ROS) is enhanced and the permeability of mitochondrial membranes increases, which results in the deterioration of mitochondrial DNA and finally to cellular apoptosis and the development of pro-inflammatory response, etc. [7,8,9,10]. The coatings of the metal core determine AgNPs’ properties, bioavailability, and compatibility with biomolecules [11]. Moreover, active research is taking place on the process of directed coating of the nanoparticle core, a process called functionalization, which is used to stabilize nanoparticles, facilitate their interaction with living tissues, and deliver them to the target.

The functionalization of nanoparticles surface with various biomolecules in a controlled manner involves a change of their properties such as prevention of aggregation or amplification of the antimicrobial effect [12,13]. However, most frequently, the main purpose of nanoparticle functionalization is to increase efficiency towards target cells and decrease toxicity towards adjacent tissues. One of the most intensely applied methods of AgNP biofunctionalization is green synthesis using biological material—extracts or microorganisms [14,15]. Biofunctionalization of AgNPs influences the level of silver accumulation in tissues but does not reduce toxic effects in relation to the target cells [16].

It was demonstrated that cyanobacteria *Spirulina platensis* can serve as a basis for AgNPs production [17]. At the same time, the biomass of spirulina itself is characterized by numerous beneficial effects on the animal and human body due to its rich and balanced biochemical content [18]. Spirulina biomass enriched with nanoparticles can be considered a very effective complex for various therapeutical purposes. However, the biosynthesis of AgNPs from silver ions is associated with the degradation of spirulina biomass as a result of the bacteriocidal effect of Ag^+^, which excludes the possibility of efficient use of such a complex. Another solution is the biofunctionalization of nanoparticles obtained by methods different from biosynthesis.

The purpose of the present study was to obtain AgNPs functionalized with spirulina biomass and to estimate the possibility of nanoparticle accumulation and excretion by organs of rats. The changes in the biochemical and hematological parameters of the rats’ blood under the influence of unmodified AgNPs versus those functionalized by biomass were assessed.

## 2. Materials and Methods

### 2.1. Nanoparticles

The polyethylene glycol coated silver nanoparticles (PEG-AgNPs) used in this study were purchased from the M9 Company (Tolyatti, Russia). Polyethylene glycol (PEG) is a polymer that is widely used to improve the stability of metal nanoparticles in medicine manufacturing and other industries.

### 2.2. Cyanobacterial Strain

*Spirulina platensis* CNMN-CB-02 from the National Collection of Nonpathogenic Microorganisms, Institute of Microbiology and Biotechnology, Moldova, was used for the production of AgNPs functionalized with spirulina biomass.

### 2.3. Production of AgNPs Functionalized with Spirulina platensis Biomass (AgNPs—Spirulina)

Cyanobacteria *Spirulina platensis*-CNMN-CB-02 were cultivated in a medium with the following composition, g/L: NaNO_3_—2.25; NaHCO_3_—8.0; NaCl—1.0; K_2_SO_4_—0.3; Na_2_HPO_4_—0.2; MgSO_4_∙7H_2_O—0.2; CaCl_2_—0.024; FeSO_4_—0.01; EDTA—0.08; H_3_BO_3_—0.00286; MnCl_2_∙4H_2_O—0.00181; ZnSO_4_∙7H_2_O—0.00022; CuSO_4_∙5H_2_O—0.00008; MoO_3_—0.000015. The following conditions were maintained with periodical stirring: pH of 9–10, temperature 28–30 °C, and illumination 37–55 µM photons/m^2^s.

On the fifth day of cultivation, which corresponds to the end of the exponential stage of growth, PEG-AgNPs in a concentration of 0.5 µM were added to the cultivation medium. The concentration was selected in a manner as to not affect significantly biomass production and its amount, which has been demonstrated in our preceding study [19]. After 24 h, the biomass was separated from the cultivation medium by filtration, standardized in distillate water, and administrated to experimental animals.

### 2.4. Extraction of the Protein Fraction from Spirulina Biomass Enriched with AgNPs

Proteins were extracted at room temperature in a buffer: 0.628 mM Tris-HCl, pH = 6.8 (1.00 g sample: 5.00 mL). In 100 mL of Tris-HCl, 5 mL of glycerin, 1 mL of β-mercaptoethanol, 0.1 g of Trilon B, and 0.5 g of 0.03% ascorbic acid were added, and the mixture was stirred for 30 min and then centrifuged for 15 min at 7000–10,000 rpm. The supernatant was collected, and the precipitate was washed twice with the buffer. Protein precipitation was performed using 50% trichloroacetic acid, up to a final concentration of 5%. The obtained precipitate was washed with ethanol, acetone, and ether and then dried.

### 2.5. Animals and Experimental Design

The experiments with animals were performed in the vivarium of the laboratory of stress physiology, adaptation, and general sanocreatology of the Institute of Physiology and Sanocreatology, Moldova, in accordance with Law No. 211/2017 of the Parliament of Republic of Moldova “On the protection of animals used for experimental or other scientific purposes” (published 05-01-2018 in *Monitorul Oficial* Nr. 1–6 art. 02), transposing Directive 2010/63/EU of the European Parliament and of the Council of 22 September 2010 on the protection of animals used for scientific purposes, published in the *Official Journal* of the European Union L 276 on 20 October 2010, and was approved by the Institutional Research Ethics Committee of the Institute of Physiology and Sanocreatology, Moldova (IREC) (IREC approval no. IREC/12/03.11.2020).

In the experiment, 24 Wistar albino rats (16 male and 8 female) were included, which were divided into four groups, consisting of 4 males and 2 females, placed in separate cages. All animals were acclimatized to standard conditions of T 22 ± 2 °C, relative humidity 55 ± 5%, and photoperiodicity 12 h light/12 h dark. During the experiment, the animals were maintained in standard conditions, with a regular diet and drinking water ad libitum. In the experimental groups, animals were supplemented as follows: 1. Negative control group (C1)—regular diet; 2. Positive control group (C2)—regular diet admixed with spirulina biomass; 3. Experimental group 1 (AgNPs)—regular diet admixed with AgNPs; 4. Experimental group 2 (AgNPs-Sp)—regular diet admixed with AgNPs-Spirulina.

The maintenance of the animals with the designated food rations was carried out for 28 days in the Vivarium of the Institute of Physiology and Sanocreatology, after which three males and one female from each group were sacrificed to collect blood for hematological and biochemical analyses and organs (brain, liver, spleen, kidneys, testicles, and ovaries) for neutron activation analysis. The remaining animals were maintained in optimal conditions, with the administration of a rationed regular diet for 28 days (clearance period) after which they were sacrificed according to the procedure described above.

The amount of AgNPs administrated to the animals in the third and fourth experimental groups was 1 µgAg/day per animal. The second group (positive control) received the same amount of spirulina biomass as the animals from the fourth experimental group. AgNPs and spirulina were incorporated into breadcrumbs from wholemeal rye flour offered in rations to rats as a first meal.

For positive control, spirulina biomass grown in the same conditions was used, but without the addition of AgNPs.

### 2.6. Methods for Nanoparticles Analysis and Silver Content Determination

#### Transmission Electron Microscopy (TEM)

The morphology of the nanoparticles was analyzed using TEM, performed using Thermo Scientific Talos F200i equipment (ThermoFisher Scientific, Waltham, MA, USA) operating at 200 kV. The TEM studies were performed at 50,000× magnification. The samples were prepared by placing a drop of silver nanoparticle solution on carbon-coated TEM grids. The films on the TEM grids were allowed to dry at room temperature before analysis.

#### Scanning Electron Microscopy (SEM)

Scanning Electron Microscopy (SEM) (FEI, Hillsboro, OR, USA) was carried out using Quanta 3D FEG. The operational features of the microscope used in the experiment were as follows: magnification of 5000–150,000×, voltage of 1–30 kV.

#### Energy-Dispersive Analysis of X-rays (EDAX)

Microprobe analysis of the silver nanoparticles was conducted with an energy-dispersive X-ray analysis spectrometer (EDAX, Waltham, MA, USA). The acquisition time ranged from 60 to 100 s, and the accelerating voltage was 20 kV.

#### Neutron Activation Analysis

The silver content in spirulina biomass and organs of rats was determined using neutron activation analysis at the IBR-2 reactor (JINR, Dubna, Russia). The description of the irradiation channels and the procedure of tissue irradiation can be found in [20,21]. Before irradiation, the samples were freeze-dried to constant weight, homogenized, and packed in aluminum bags.

The tissue samples were irradiated with thermal neutrons for 3 days at a neutron flux of 1.2 × 10^11^ cm^−2^ s^−1^, repacked, and measured for 1.5 h. Gamma spectra of induced activity were measured by using three spectrometers based on HPGe detectors with an efficiency of 100% and resolution of 1.8–2.0 keV for the 1332 keV total-absorption peak of the isotope ^60^Co. The analysis of the spectra was performed by using the Genie2000 software (Canberra, Zellik, Belgium), with verification of the peak fit in an interactive mode. Calculation of the concentrations was carried out using the software “Concentration” developed in FLNP. Quality control of the analytical results was provided by comparing the calculated and certified (passport) concentrations for the standard reference materials: NIST 2710a (Montana I Soil Highly Elevated Trace Element Concentrations) and liquid Ag standard (Merk, Darmstadt, Germany). The difference between calculated and certified values did not exceed 5%.

### 2.7. Blood Hematology and Biochemistry

Biochemical analysis of the blood was performed by using a semi-automated photometer StarDust MC15 (DiaSys Diagnostic Systems, Holzheim, Germany). Hematological analysis used an automated hematology analyzer, Sysmex XT-2000i Hematology Analyzers (GMI Inc, Ramsey, MN), USA), by using the default analysis settings.

### 2.8. Statistical Analysis

All experiments were performed in triplicate. Thus, for males from each group, 4 × 3 = 12 tests were performed, and 2 × 3 = 6 tests were performed for females. The results in all tables and histograms are presented as mean values ± standard deviations. Differences between the values were estimated by Student’s *t*-tests.

## 3. Results

### 3.1. Nanoparticle Characterization

**Figure 1:**
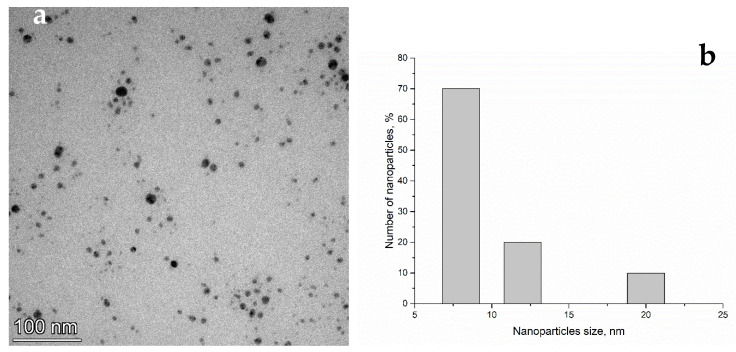
TEM image (**a**) and size histogram (**b**) of AgNPs nanoparticles.

The morphology of the AgNPs and their size distribution are presented in Figure 1. The TEM image taken of the drop-cast film of AgNPs dissolved in water shows that the nanoparticles were mainly spherical and formed small agglomerations. The size of the nanoparticles ranged from 8 nm to 20 nm. More details about AgNP characterization can be found in our previous study [19].

### 3.2. Characterization of AgNPs-Spirulina and Determination of the Concentration of Nanoparticles Administered to Animals

Spirulina biomass containing AgNPs was analyzed in order to determine silver content in the biomass and its biochemical composition. According to the performed analysis, the biomass contained 64.7 ± 2.4% proteins, 4.6 ± 0.6% lipids, 11.5 ± 0.8% carbohydrates, 0.26 ± 0.05% β-carotene, and 12.4 ± 1.1% phycobiliproteins. All biochemical parameters were on the level of the control biomass (cultivated without the addition of nanoparticles) (see Table S1). The content of silver in the biomass determined by NAA was 51 µg/g. Thus, in order to receive 1 µg of silver, each animal was administrated with 20 mg of AgNPs-Spirulina. Animals from the positive control group were administrated 20 mg of spirulina biomass without nanoparticles.

**Figure 2:**
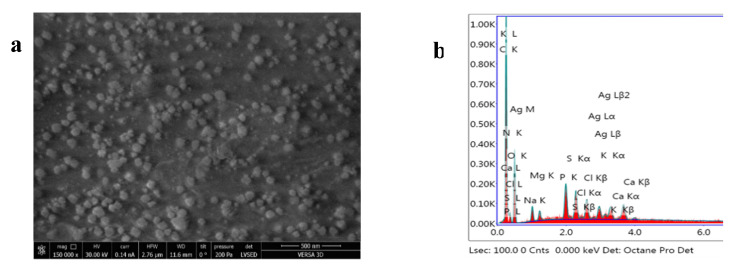
AgNPs-Spirulina (protein fraction) **a**. SEM; **b**. EDAX.

In order to confirm nanoparticle trapping to the spirulina biomass, the protein fraction, which was 0.52 g of protein per gram of biomass, was extracted. The content of silver in the protein fraction was 41 µg/g, meaning that about 80.4% of the total amount of silver was accumulated in the biomass. The SEM images and EDAX spectrum obtained for the AgNPs-Spirulina biomass are presented in Figure 2.

### 3.3. Biological Parameters of Animals and Organs after AgNPs and AgNPsSp Administration

During the experiment, the rats in all groups gained weight, and no visible differences were observed between them. Thus, males increased their body mass by 50–55% over 28 days, and females increased their body mass by 52–56%. No differences in the macroscopic and histological appearances of the collected organs were detected. There were also no significant differences in terms of the mass of organs (Table S2).

### 3.4. Content of Silver in Animal Organs Following Administration of AgNPs and after a Period of Clearance

**Figure 3:**
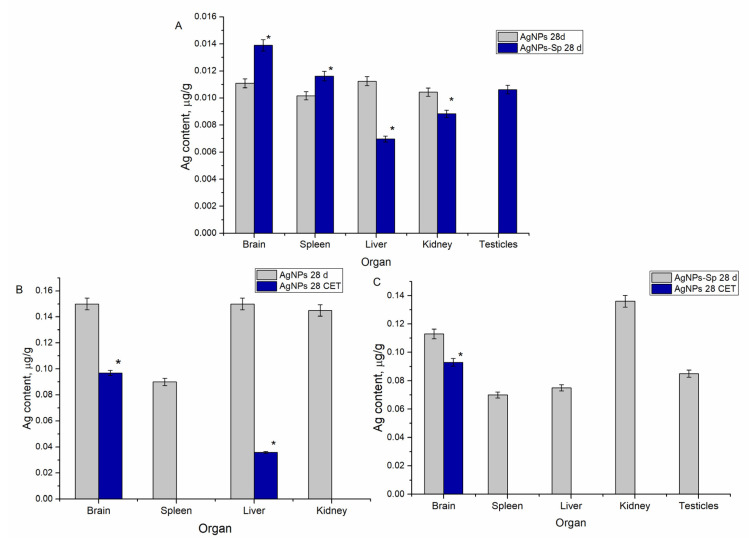
The content of silver in rats’ organs: (**A**) animals administrated with AgNPs and AgNPs-Spirulina for 28 days, measured immediately after the end of the experiment; (**B**) animals administrated with AgNPs for 28 days with measurements after a clearance period; (**C**) animals administrated with AgNPs-Spirulina for 28 days after a clearance period. 28 CET—clearance expiry time of 28 days. * indicates *p* ˂ 0.005 for the difference between neighboring groups.

The silver content determined in the organs of the laboratory animals is shown in Figure 3. As observed from Figure 3A, silver was detected in all animal organs, except in the ovarian and testicular glands in which only functionalized nanoparticles accumulated. The highest content of silver was determined in the brain, liver, and kidney samples—0.145–0.150 µg/g.

The administration of AgNP-Spirulina produced results different from those obtained for animals administrated with unmodified nanoparticles. The highest content of silver was determined in the brain and kidneys, while the amounts of silver accumulated in the spleen and liver were almost on the same level. In the case of the brain and liver, the amount of metal accumulated is significantly lower compared to the results for unmodified nanoparticles. It is important to mention that testicles accumulated considerable amounts of silver from AgNP-Spirulina, comparable with the content in the spleen and liver.

Figure 3B shows the results related to the excretion of unmodified AgNPs from animal organs after 28 days of clearance. According to obtained values, silver was eliminated from the spleen and kidneys, and the amount in the liver was reduced by 76%. A different scenario was observed in the case of the brain, where silver content remained on a relatively high level. Although the difference in silver content before and after the clearance period was statistically significant, only 35% of the accumulated metal was removed from the brain. Figure 3C demonstrates the results obtained after the clearance period in the case of regular diet admixed with AgNPs-Spirulina nanoparticles. Silver was completely removed from the spleen, liver, kidneys, and testicles, but it remained in the brain where its level did not differ statistically from the values obtained immediately after the administration period.

### 3.5. Hematological Test Results for Laboratory Animals Administrated with AgNPs

**Figure 4:**
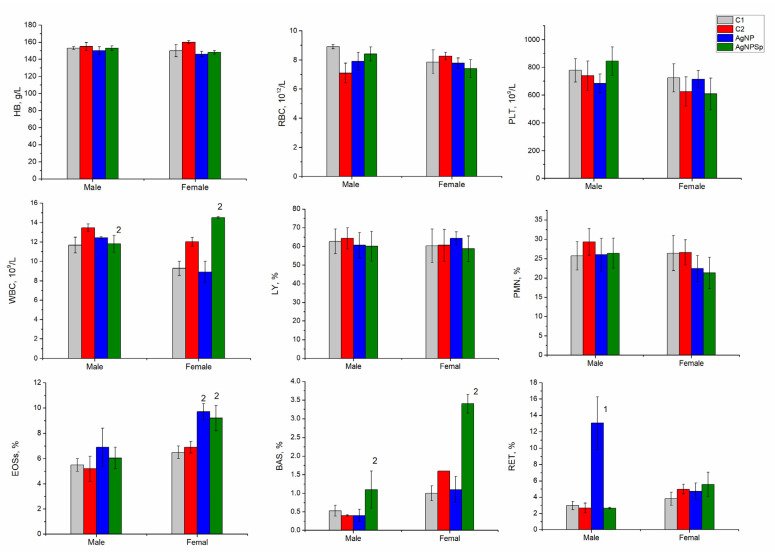
Hematological indices of rats’ blood. C1—negative control; C2—positive control (spirulina); AgNPs—experimental group administrated with AgNPs; AgNPsSp—experimental group administrated with AgNPs-Spirulina. HB—hemoglobin; RBC—red blood cells; WBC—white blood cells; PLT—platelet count; PMN—polymorphomultinuclear neutrophil granulocytes; LY—lymphocytes; EOS—eosinophils; BAS—basophils; RET—reticulocytes. 1: *p* ˂ 0.005 for differences between the AgNPs and C1 groups; 2: *p* ˂ 0.005 for differences between the AgNPs-Sp and C2 groups.

Figure 4 reflects the results obtained in hematological tests for animals from the four groups at the end of the 28-day of nanoparticle administration, performed immediately after the sacrificing the animals.

From the figure, it is observed that the amount of hemoglobin and RBC did not change depending on the type of administered nanoparticles and the sex of the animals, and the values obtained for males and females were at the level of the negative control. No significant changes in platelet content (PLT) were detected, and oscillations in the PLT content observed in females were within the limit of normal physiological values. WBC values were within normal value characteristics for laboratory rats, and some oscillations depend on the animals’ sex. At administration with AgNPs-Spirulina, the number of leukocytes increased statistically significantly compared to the control values, but they did not exceed the values accepted as normal for rats [22,23].

The ratio of LY and PMN did not change; thus, the obvious inflammatory response that could be attributed to the toxic effect of nanoparticles was lacking. Instead, some immune responses such as allergic reactions were established in the female group, in which, as a result of AgNP administration, the content of eosinophils increased (EOSs—9.7% of leukocytes for AgNP and 9.2% for AgNPs-Spirulina). The difference in the content of eosinophils in the experimental and control groups was statistically significant.

### 3.6. Biochemical Test Results for Laboratory Animals Administrated with AgNPs

**Figure 5:**
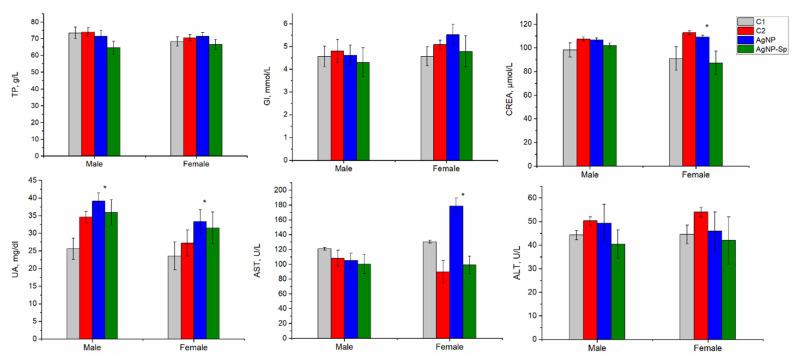
Biochemical indices of blood serum in rats. C1—negative control; C2—positive control (spirulina); AgNPs—experimental group administrated with AgNPs; AgNPsSp—experimental group administrated with AgNPs-Spirulina. TP—total proteins; GL—glucose; CREA—creatinin; UA—urea; ALT—alanine aminotransferase; AST—aspartate aminotransferase; (n male = 4, n tests = 12; n female = 2, n tests = 6). *—*p* ˂ 0.005 for AgNPs and C1 groups.

The results of the biochemical tests for animals from all four groups, performed immediately after sacrificing the animals at the end of the 28-day of nanoparticle administration, are presented in Figure 5.

In the present study, standard inflammatory biochemical parameters such as alanine aminotransferases (ALT), aspartate aminotransferases (AST), total proteins (TP), glucose (GL), urea (UA), and creatinine (CREA) were determined. The content of total proteins and glucose did not change in the animals of the experimental groups, regardless of the type of nanoparticles administered or animals’ sex. Although the liver accumulated significant amounts of silver, the indicators of liver function, ALT and AST, did not differ significantly between groups. In the case of females, in the group that received unmodified nanoparticles, a high level of AST was observed, although it laid within the limits of normal values. An increase in UA values was observed in three of the experimental variants compared to the negative control, but the values did not exceed normal limits, rather they were at the upper limit of the normal values. This may indicate an overload of the kidneys caused by additional protein intake from spirulina biomass and/or minor toxic effect of AgNPs.

### 3.7. Hematological and Biochemical Indicators in Laboratory Animals at the End of the Clearance Period

The results of hematological and biochemical tests performed after the clearance period are presented in Table 1. The main part of hematological and biochemical indicators did not change, as expected, and the values determined at the end of the period of nanoparticle administration were within the reference values. However, some differences can be noted. At the expiration of the clearance period, in the experimental group with AgNPs administration, a normalization of the number of eosinophils was established. For the group supplemented with food-admixed AgNP-Spirulina, the content of eosinophils continued to increase and the value was almost twice higher than in the group with AgNPs administration. The animals remained sensitized to the AgNPs-Spirulina complex. It is worth mentioning the restoration of basophil content, which initially increased when animals were administered with AgNPs-Spirulina.

In the same experimental variant, after the clearance period, a significant increase in the content of ALT was observed. Since ALT is an indicator specific to liver function, increased activity of this enzyme may be directly related to the delayed toxic effect of nanoparticles on the liver. An increase in glucose content in blood at the end of the clearance period was observed. The values obtained did not exceed the reference values but were at the upper limit, which indicated a persistent weak immune response.

## 4. Discussion

The main aim of the present study was to obtain biofunctionalized AgNPs using spirulina culture and to compare their effects on rats with respect to AgNPs stabilized by polyethylene glycol. In order to obtain AgNPs-Sp, a new approach, different from those described in specialized literature, was applied. For this purpose, was used spirulina biomass at the stage of exponential growth, and engineered nanoparticles at low concentration as a material for biofunctionalization were used, which did not affect the properties of spirulina. As a result, the obtained biomass contained proteins, carbohydrates, lipids, and pigments on levels similar to the control biomass (see Table S1). Nanoparticle accumulation by spirulina biomass was confirmed by two techniques. Applying NAA, the content of silver in spirulina biomass was ascertained, while SEM and EDAX techniques allowed visualization and identification of AgNPs in the proteinic fraction. The results revealed that at least 80.4% of the AgNPs captured by the spirulina biomass were biofunctionalized with the protein fraction. This approach, applied in the biofunctionalization of the nanoparticles, allowed obtaining AgNPs with properties different from the unmodified ones, as well as benefitting from the advantageous biological properties of the spirulina biomass.

According to literature data, experiments on AgNP biofunctionalization are based on silver ions and the application of different biological systems used as a matrix for biofunctionalization: bacteria, fungi, plants extracts, biological liquids, and biomolecules such as vitamins, amino acids, and enzymes. The principle of these techniques consists in the use of biological components as reducing agents for silver ions and the investigation of the newly acquired biological effects of the biofunctionalized nanoparticles, as it is known that they differ depending on the applied biological matrix [24,25,26].

In previously performed studies, the nanoparticles obtained by green synthesis were separated from the biological matrix in which they were incorporated, which most often resulted in severe biomass damage. As demonstrated in our study, during AgNP production from silver nitrate, the content of proteins in the spirulina biomass was reduced by 22.7–71.7%, by 84% with respect to carbohydrates, and by 26.5–62% with respect to of lipids [17].

The cyanobacterium *Spirulina platensis* has a distinct biological value, as it possesses the ability to modify the toxicity and bioavailability of nanoparticles, to attenuate or amplify their biological effects, and in addition, it is a suitable matrix for nanoparticle production. Thus, an active complex consisting of biofunctionalized nanoparticles and components of spirulina biomass was obtained, which essentially distinguishes this study from other research.

The biological effects of nanoparticles have been studied extensively in the last years, and laboratory animals, more often rats, are used as models. In the present study, the accumulation of both unmodified and biofunctionalized with spirulina nanoparticles in various organs, including reproductive ones, was assessed. Both types of nanoparticles were accumulated in the liver, spleen, kidneys, and brain; however, the level of nanoparticle accumulation was organ-specific. Thus, in the brain and spleen, the amount of accumulated silver at the administration of biofunctionalized nanoparticles was significantly higher compared to the unmodified nanoparticles. In the liver and kidneys—the organs responsible for excretion and detoxification of administered substances—the situation was reversed, and silver accumulation was more pronounced in the case of unmodified nanoparticles. It is interesting to mention that ovaries did not accumulate silver regardless of the form of nanoparticles administered, whereas testicles accumulated the metal in significant quantities (comparable to the spleen) only in the case of administering with AgNPs-Sp.

The main part of the available research involving laboratory animals is focused on the administration of relatively high concentrations of nanoparticles (as compared to our investigation)—tens of mg per day per animal. In these conditions, high concentrations of silver were determined in the ileum, liver, kidney, brain, thymus, and spleen. The accumulation of silver in various organs did not have a specific dose-dependent character in these studies. For example, as the dose of nanoparticles increases, the amount of silver accumulated in the liver increases; but in the brain, the dose increases to a certain threshold concentration, yet at the administration of even higher AgNP concentrations, it decreases. When high doses of nanoparticles are administered, the highest concentrations of silver were accumulated in the liver, kidneys, and spleen [27,28,29,30]. These results are in agreement with data obtained in the present study: significant accumulation of silver in the liver and kidneys, as well as in the brain and spleen, even at low AgNP concentration (28 µg of Ag for 28 days).

Previously, the accumulation of silver in testicles was ascertained when animals were administrated with nanoparticles biofunctionalized with the fungus *Gibberella sp.* [30], which is a result similar to the data in the present study. In our study, at the end of the clearance period, silver was not detected in the testicles. According to obtained and literature data, testicles are an important target for the accumulation of AgNPs, both unmodified and functionalized by spirulina biomass. This can not only be explained by the affinity of the tissues to AgNPs but also relative to the components of the spirulina biomass. This property of the AgNPs-Sp complex can be further explored for practical purposes in the development of targeted drugs.

The amount of silver accumulated in the brain was quite high; thus, results and data presented by other authors confirmed that AgNPs are able to penetrate the blood–brain barrier. This fact is extremely important because it relates to a potential risk for humans. In the conditions of ever-increasing use of AgNPs in various industries, including food packaging, they can easily enter the digestive tract and be transported to the brain. This risk becomes even more evident in our study given that, after a clearance period of 28 days, only 35% of the accumulated silver was removed from the brains of the animals. The amount of silver that remained in the brain was approximately equal for both types of nanoparticles. Other authors have also established the persistence of silver accumulated in the brain even after a period of clearance, highlighting its slow elimination from this organ [31].

Some authors showed that the accumulation of silver in the organs of rats depends on their sex [32]. Thus, at AgNPs doses of 30, 300, and 1000 mg/kg/day, the highest concentrations of silver were determined in the liver and kidneys of female rats. In the present study, such dependence was not observed, but it should be noted that the concentration of administered nanoparticles was significantly lower.

In the main part of the research, the liver was defined as an organ with the maximum accumulation of AgNPs, regardless of the mode and dose of nanoparticle administration. The hepatotoxicity of AgNPs can be explained by the alteration of the activity of liver enzymes. In the present study, at the administration of AgNPs in a dose of 1µg/animal/day for 28 days, an increase in ALT values in females was determined compared to the negative control. An increase in AST values was observed in the group of animals administrated with AgNPs-Sp and subjected to a clearance period, although after the clearance period in this group, the removal of silver from the liver took place. Thus, the accumulation of silver in the liver as a result of the administration of AgNPs is not necessarily accompanied by changes in liver indicators. Similar results have been obtained by other researchers [29,32,33]. For example, an increase in ALT and a decrease in protein content were observed when rats were administered 60 nm AgNPs in doses of 30, 300, and 100 mg kg/day for 28 days [32]. Chitosan coated AgNPs induced dose-dependent toxicity with the highest levels of ALT and AST achieved for the maximum dose used in the study—50 mg/kg body weight [34]. Another biochemical indicator of liver tissue damage is glucose levels [35]. The glucose levels (Figure 5) at the upper limit in both experimental groups support this fact. It is important to note that high glucose values were recorded at the end of the clearance period, which may indicate delayed or long-lasting effects.

Some authors attest to an increase in renal markers, such as creatinine [29], which correlates with our results obtained in the case of unmodified nanoparticles and positive control in females. During performed experiments, another marker—urea—is reported to increase with the administration of nanoparticles to rats of both sexes.

The accumulation of silver in different organs as a result of the administration of AgNP of different sizes, at different doses, and in different ways induce the development of the pro-inflammatory immune response. In the present study, due to the accumulation of AgNPs in rats, the content of eosinophil increased up to 9.7% of leukocytes for AgNPs and up to 9.2% for AgNPs-Spirulina. The sensitization produced is also confirmed by the basophil content (BAS), which increased significantly from 1% of leukocytes in the negative control variant to 3.4% in the experimental variant of AgNPs-Spirulina administration in females. Thus, sensitization of the immune system by increasing the content of eosinophils and basophils was observed only in females, while a significant increase in reticulocyte content (RET) in the blood was noticed in males at the administration with unmodified nanoparticles, which is 13% of leukocytes, compared to values up to 6% considered as normal [36]. The increased content of reticulocytes may be considered as an immune response or an accommodative reaction. Some authors consider the increase in the number of reticulocytes as a hematological reaction to the decrease in hemoglobin [34], although it was not observed in the performed study.

Although the main part of the reviewed studies does not report severe changes in hematologic indicators as a result of the administration of AgNPs [33,37], in some cases deviations from the norm are observed, such as an increase in the number of neutrophils in blood as a result of administration with 20 nm AgNP [33] or an increase in the number of RBCs in males and a decrease in the number of PLTs in both males and females when taking AgNP at doses of 0.5–1 mg/kg for 28 days [17]. The obtained results denote some deviations from the values recorded in the control group, such as the change of WBC at the administration of AgNPs-Sp compared to the positive control and the increase in the number of basophils and eosinophils. After the clearance period, most of these parameters showed values not significantly different from the control.

Thus, the results obtained regarding the effects on different hematological and biochemical parameters in laboratory animals suggest the need for further research in this field. The application of complex approaches is necessary in order to highlight the regularities arising from the interaction of AgNPs with a living organism.

## 5. Conclusions

1. Spirulina biomass can be used for the biofunctionalization of AgNPs stabilized with polyethylene glycol, as approximately 80% of them were trapped to the proteinic fraction of biomass.
2. Both types of nanoparticles (biofunctionalized using spirulina culture and AgNPs stabilized by polyethylene glycol) were accumulated in the brain, spleen, liver, and kidneys. Biofunctionalized particles showed a higher affinity for the brain and spleen, whereas the unmodified ones showed higher affinity for the liver and kidneys.
3. There was no accumulation of nanoparticles in the ovaries, while in the testicles biofunctionalized nanoparticles were accumulated only. This selectivity can serve as a basis for the development of preparations based on targeted AgNPs.
4. During the clearance period, silver in the form of AgNPs-Spirulina was excreted from all organs, except the brain (where 82.3% of accumulated content remained), whereas unmodified AgNPs were excreted completely from the spleen and kidneys, but 24% of the silver accumulated in the liver and 65% in the brain remained. Thus, both types of studied AgNPs easily crossed the blood–brain barrier in the direction of the brain, while the reverse flow was very low.
5. Based on the level of silver accumulation and elimination from the kidneys and liver, the involvement of these two organs in AgNPs elimination and the preferential renal pathway in the case of AgNPs-Spirulina can be assumed.
6. The hematological and biochemical parameters of blood in rats after administration of silver nanoparticles remained within the normal parameters for the species; however, statistically significant differences were observed for some of them.
7. The change in the level of eosinophils, basophils, and reticulocytes indicated moderate immune reactions.
8. The changes in the renal and hepatic parameters pointed to the probable toxicity of the studied nanoparticles relative to these organs.
9. AgNPs should be used with caution by taking into account their persistence in the brain and evidence for some delayed or prolonged effects over time.

## Figures and Tables

**Table 1:** Hematological and biochemical indicators in rats after 28 days of AgNPs administration and a clearance period of 28 days.

	AgNPs 28d	AgNPs CET	AgNPs-Sp 28d	AgNPs-Sp CET
HB, g/L	149 ± 4.67	148 ± 0.71	152 ± 0.67	148.5 ± 7.78
RBC, 10^12^/L	7.77 ± 0.11	8.07 ± 0.77	8.4 ± 0.61	7.99 ± 0.68
WBC, 10^9^/L	10.82 ± 1.28	15.69 ± 3.98	11.72 ± 1.92	10.16 ± 1.7
PLT, 10^9^/L	647.3 ± 68.2	886.5 ± 7.78	812.6 ± 106.2	730.0 ± 46.67
PMN, %	25.07 ± 2.49	48.25 ± 4.74	24.0 ± 2.81	33.7 ± 8.34
LY %	61.83 ± 3.49	36.35 ± 0.71	59.63 ± 1.51	49.7 ± 4.53
EOS, %	8.3 ± 1.98	4.1 ± 0.05 *	7.60 ± 2.23	12.45 ± 5.45
BAS, %	0.75 ± 0.13	0.25 ± 0.07 *	2.25 ± 1.63	0.35 ± 0.071 *
RET, %	8.9 ± 5.3	2.95 ± 0.73	4.1 ± 2.05	2.93 ± 0.69
TP, g/L	75.17 ± 10.02	71.15 ± 11.19	65.03 ± 4.52	73.8 ± 9.48
GL, mmol/L	4.83 ± 0.61	6.87 ± 0.5 *	4.42 ± 0.54	6.06 ± 0.83 *
CREA, µmol/L	107.3 ± 12.42	105.5 ± 5.09	98.25 ± 7.3	98.25 ± 5.16
UA, mg/dL	37.67 ± 6.67	35.48 ± 1.85	34.82 ± 8.53	37.67 ± 2.47
ALT, U/L	47.65 ± 2.35	50.95 ± 10.21	41.15 ± 1.20	220.6 ± 42.3 *
AST, U/L	141.66 ± 52.08	66.6 ± 8.72	99.6 ± 0.99	115.35 ± 29.3

* *p* ˂ 0.005 for the differences between the groups AgNPs/AgNPs-Sp 28d and AgNPs/AgNPs-Sp CET).

## Supplementary Materials

The following are available online at https://www.mdpi.com/article/10.3390/nano11112992/s1, Table S1: Biochemical parameters of *Spirulina platensis* cultivated with and without addition of AgNPs, Table S2: Mass of the rats’ organs collected at the end of 28-day experiments (g).

## References

[1] Parveen S., Misra R., Sahoo S.K. (2012). Nanoparticles: A boon to drug delivery, therapeutics, diagnostics and imaging. Nanomed. Nanotechnol. Biol. Med..

[2] Calderón-Jiménez B., Johnson M.E., Montoro Bustos A.R., Murphy K.E., Winchester M.R., Baudrit J.R.V. (2017). Silver nanoparticles: Technological advances, societal impacts, and metrological challenges. Front. Chem..

[3] Keat C.L., Aziz A., Eid A.M., Elmarzugi N.A. (2015). Biosynthesis of nanoparticles and silver nanoparticles. Bioresour. Bioprocess..

[4] Zhang X.F., Liu Z.G., Shen W., Gurunathan S. (2016). Silver nanoparticles: Synthesis, characterization, properties, applications, and therapeutic approaches. Int. J. Mol. Sci..

[5] Bruna T., Maldonado-Bravo F., Jara P., Caro N. (2021). Silver nanoparticles and their antibacterial applications. Int. J. Mol. Sci..

[6] Liao S., Zhang Y., Pan X., Zhu F., Jiang C., Liu Q., Cheng Z., Dai G., Wu G., Wang L. (2019). Antibacterial activity and mechanism of silver nanoparticles against multidrug-resistant Pseudomonas aeruginosa. Int. J. Nanomed..

[7] Almofti M.R., Ichikawa T., Yamashita K., Terada H., Shinohara Y. (2003). Silver ion induces a cyclosporine A-insensitive permeability transition in rat liver mitochondria and release of apoptogenic cytochrome c. J. Biochem..

[8] Antony J.J., Sivalingam P., Chen B. (2015). Toxicological effects of silver nanoparticles. Environ. Toxicol. Pharmacol..

[9] Haberl N., Hirn S., Wenk A., Diendorf J., Epple M., Johnston B.D., Krombach F., Kreyling W.G., Schleh C. (2013). Cytotoxic and proinflammatory effects of PVP-coated silver nanoparticles after intratracheal instillation in rats. Beilstein J. Nanotechnol..

[10] Rahman M.F., Wang J., Patterson T.A., Saini U.T., Robinson B.L., Newport G.D., Murdock R.C., Schlager J.J., Hussain S.M., Ali S.F. (2009). Expression of genes related to oxidative stress in the mouse brain after exposure to silver-25 nanoparticles. Toxicol. Lett..

[11] Monopoli M.P., Bombelli F.B., Dawson K.A. (2011). Nanoparticle coronas take shape. Nat. Nanotechnol..

[12] Le Ouay B., Stellacci F. (2015). Antibacterial activity of silver nanoparticles: A surface science insight. Nano Today.

[13] Ravindran A., Chandran P., Khan S.S. (2013). Biofunctionalized silver nanoparticles: Advances and prospects. Colloids Surf. B Biointerfaces.

[14] Dinparvar S., Bagirova M., Allahverdiyev A.M., Abamor E.S., Safarov T., Aydogdu M., Aktas D. (2020). A nanotechnology-based new approach in the treatment of breast cancer: Biosynthesized silver nanoparticles using Cuminum cyminum L. seed extract. J. Photochem. Photobiol. B Biol..

[15] Gurunathan S., Raman J., Abd Malek S.N., John P.A., Vikineswary S. (2013). Green synthesis of silver nanoparticles using Ganoderma neo-japonicum Imazeki: A potential cytotoxic agent against breast cancer cells. Int. J. Nanomed..

[16] Opris R., Toma V., Olteanu D., Baldea I., Baciu A.M., Lucaci F.I., Berghian-Sevastre A., Tatomir C., Moldovan B., Clichici S. (2019). Effects of silver nanoparticles functionalized with Cornus mas L. extract on architecture and apoptosis in rat testicle. Nanomedicine.

[17] Cepoi L., Rudi L., Chiriac T., Valuta A., Zinicovscaia I., Duca G.H., Kirkesali E., Frontasyeva M., Culicov O., Pavlov S. (2014). Biochemical changes in cyanobacteria during the synthesis of silver nanoparticles. Can. J. Microbiol..

[18] Furmaniak M.A., Misztak A.E., Franczuk M.D., Wilmotte A., Waleron M., Waleron K.F. (2017). Edible cyanobacterial genus Arthrospira: Actual state of the art in cultivation methods, genetics, and application in medicine. Front. Microbiol..

[19] Cepoi L., Zinicovscaia I., Rudi L., Chiriac T., Rotari I., Turchenko V., Djur S. (2020). Effects of PEG-coated silver and gold nanoparticles on spirulina platensis biomass during its growth in a closed system. Coatings.

[20] Zinicovscaia I., Pavlov S.S., Frontasyeva M.V., Ivlieva A.L., Petritskaya E.N., Rogatkin D.A., Demin V.A. (2018). Accumulation of silver nanoparticles in mice tissues studied by neutron activation analysis. J. Radioanal. Nucl. Chem..

[21] Frontasyeva M.V. (2008). Epithermal Neutron activation analysis at the ibr-2 reactor of the frank laboratory of neutron physics at the joint institute for nuclear research (Dubna). Phys. At. Nucl..

[22] Delwatta S.L., Gunatilake M., Baumans V., Seneviratne M.D., Dissanayaka M.L.B., Batagoda S.S., Udagedara A.H., Walpola P.B. (2018). Reference values for selected hematological, biochemical and physiological parameters of Sprague-Dawley rats at the Animal House, Faculty of Medicine, University of Colombo, Sri Lanka. Anim. Model. Exp. Med..

[23] Wolford S.T., Schroer R.A., Gohs F.X., Gallo P.P., Brodeck M., Falk H.B., Ruhren R. (1986). Reference range data base for serum chemistry and hematology values in laboratory animals. J. Toxicol. Environ. Health.

[24] Das B., Tripathy S., Adhikary J., Chattopadhyay S., Mandal D., Dash S.K., Das S., Dey A., Dey S.K., Das D. (2017). Surface modification minimizes the toxicity of silver nanoparticles: An in vitro and in vivo study. JBIC J. Biol. Inorg. Chem..

[25] Garza-Ocañas L., Ferrer D.A., Burt J., Diaz-Torres L.A., Ramírez Cabrera M., Rodríguez V.T., Rangel R.L., Romanovicz D., Jose-Yacaman M. (2010). Biodistribution and long-term fate of silver nanoparticles functionalized with bovine serum albumin in rats. Metallomics.

[26] Gurunathan S., Jeong J.-K., Han J.W., Zhang X.-F., Park J.H., Kim J.-H. (2015). Multidimensional effects of biologically synthesized silver nanoparticles in Helicobacter pylori, Helicobacter felis, and human lung (L132) and lung carcinoma A549 cells. Nanoscale Res. Lett..

[27] Garcia T., Lafuente D., Blanco J., Sánchez D.J., Sirvent J.J., Domingo J.L., Gómez M. (2016). Oral subchronic exposure to silver nanoparticles in rats. Food Chem. Toxicol..

[28] Loeschner K., Hadrup N., Qvortrup K., Larsen A., Gao X., Vogel U., Mortensen A., Lam H.R., Larsen E.H. (2011). Distribution of silver in rats following 28 days of repeated oral exposure to silver nanoparticles or silver acetate. Part. Fibre Toxicol..

[29] Wen H., Dan M., Yang Y., Lyu J., Shao A., Cheng X., Chen L., Xu L. (2017). Acute toxicity and genotoxicity of silver nanoparticle in rats. PLoS ONE.

[30] Qin G., Tang S., Li S., Lu H., Wang Y., Zhao P., Li B., Zhang J., Peng L. (2017). Toxicological evaluation of silver nanoparticles and silver nitrate in rats following 28 days of repeated oral exposure. Environ. Toxicol..

[31] Recordati C., De Maglie M., Cella C., Argentiere S., Paltrinieri S., Bianchessi S., Losa M., Fiordaliso F., Corbelli A., Milite G. (2021). Repeated oral administration of low doses of silver in mice: Tissue distribution and effects on central nervous system. Part. Fibre Toxicol..

[32] Kim Y.S., Kim J.S., Cho H.S., Rha D.S., Kim J.M., Park J.D., Choi B.S., Lim R., Chang H.K., Chung Y.H. (2008). Twenty-eight-day oral toxicity, genotoxicity, and gender-related tissue distribution of silver nanoparticles in Sprague-Dawley rats. Inhal. Toxicol..

[33] Hassanen E.I., Khalaf A.A., Tohamy A.F., Mohammed E.R., Farroh K.Y. (2019). Toxicopathological and immunological studies on different concentrations of chitosan-coated silver nanoparticles in rats. Int. J. Nanomed..

[34] De Jong W.H., Van Der Ven L.T.M., Sleijffers A., Park M.V.D.Z., Jansen E.H.J.M., Van Loveren H., Vandebriel R.J. (2013). Systemic and immunotoxicity of silver nanoparticles in an intravenous 28 days repeated dose toxicity study in rats. Biomaterials.

[35] Dasgupta N., Ranjan S., Ramalingam C., Gandhi M. (2019). Silver nanoparticles engineered by thermal co-reduction approach induces liver damage in Wistar rats: Acute and sub-chronic toxicity analysis. 3 Biotech.

[36] Torous D.K., Avlasevich S.L., Khattab M.G., Baig A., Saubermann L.J., Chen Y., Bemis J.C., Lovell D.P., Walker V.E., MacGregor J.T. (2020). Human blood PIG-A mutation and micronucleated reticulocyte flow cytometric assays: Method optimization and evaluation of intra- and inter-subject variation. Environ. Mol. Mutagen..

[37] Yang L., Kuang H., Zhang W., Aguilar Z.P., Wei H., Xu H. (2017). Comparisons of the biodistribution and toxicological examinations after repeated intravenous administration of silver and gold nanoparticles in mice. Sci. Rep..

