# Supplementary material for: Accumulation and Effect of Silver Nanoparticles Functionalized with Spirulina platensis on Rats
Source: Nanomaterials (Basel). 2021 Nov 7;11(11):2992. doi: 10.3390/nano11112992 (PMC8620753; doi:10.3390/nano11112992)
Supplement: Supplementary file 1 [file nanomaterials-11-02992-s001.zip › nanomaterials-1420410-supplementary.pdf]

Article

Accumulation and Effect of Silver Nanoparticles Functionalized with *Spirulina platensis* on Rats

Ludmila Rudi [1], Inga Zinicovscaia [2,3,4,*], Liliana Cepoi [1], Tatiana Chiriac [1], Alexandra Peshkova [2], Anastasia Cepoi [1] and Dmitrii Grozdov [2]

[1] Institute of Microbiology and Biotechnology, 1 Academiei Str., 2028 Chisinau, Moldova; rudiludmila@gmail.com (L.R.); lilianacepoi@yahoo.com (L.C.); chiriac.tv@gmail.com (T.C.); anastasiacepoi@gmail.com (A.C.)
[2] Joint Institute for Nuclear Research, 6 Joliot-Curie Str., 141980 Dubna, Russia; peshkova.alexandra92@gmail.com (A.P.); dsgrozdov@rambler.ru (D.G.)
[3] Horia Hulubei National Institute for R&D in Physics and Nuclear Engineering, 30 Reactorului Str. MG-6, Bucharest Magurele, Romania
[4] Institute of Chemistry, 3 Academiei Str., 2028 Chisinau, Moldova
* Correspondence: zinikovskaia@mail.ru; Tel.: +7-4962165609

Table S1. Biochemical parameters of Spirulina platensis cultivated with and without addition of AgNPs.

S. platensis	**Biomass, g/L**	**Proteins, %**	**Carbohydrates, %**	**Lipids, %**	**β-carotene, %**	**Phycobiliproteins, %**
Control	0.98 ± 0.11	67.4 ± 0.69	10.57 ± 0.12	4.8 ± 0.072	0.31±0.06	14.2±0.98
+ AgNPs	1.03 ± 0.026	64.7 ± 2.40	11.50 ± 0.80	4.6 ± 0.600	0.26± 0.05	12.4±1.10

Table S2. Mass of the rats' organs collected at the end of 28-day experiments (g).

		**Brain**	**Liver**	**Spleen**	**Kidney**	**Ovaries**	**Testicles**
C1	M	1.57 ± 0.22	8.22 ± 0.7	0.67 ± 0.12	1.67 ± 0.09		2.45 ± 0.17
	F	1.62 ± 0.07	7.7 ± 0.8	0.8 ± 0.4	1.4 ± 0.28	0.14 ± 0.05	
C2	M	1.6 ± 0.08	8.1 ± 1.2	0.6 ± 0.14	1.55 ± 0.1		2.1 ± 0.4
	F	1.55 ± 0.21	7.2 ± 0.9	0.8 ± 0.4	1.4 ± 0.14	0.15 ± 0.07	
AgNPs	M	1.7 ± 0.08	8.45 ± 0.9	0.7 ± 0.18	1.7 ± 0.2		2.65 ± 0.1
	F	1.6 ± 0.14	7.2 ± 0.3	0.75 ± 0.14	1.5 ± 0.14	0.15 ± 0.07	
AgNPs-Sp	M	1.57 ± 0.15	8.3 ± 1.8	0.63 ± 0.15	1.73 ± 0.18		2.43 ± 0.15
	F	1.55 ± 0.07	7.0 ± 0.7	0.8 ± 0.07	1.45 ± 0.2	0.14 ± 0.05	
